# Analysis of Literary Situation and Reconstruction of the Writing Subject in Literary Education by Educational Psychology

**DOI:** 10.3389/fpsyg.2021.727413

**Published:** 2022-01-06

**Authors:** Gaonan Xu, Zhaoming Li, Fengrui Zhang, Bojing Liu

**Affiliations:** ^1^School of Translation Study, Shandong University, Weihai, China; ^2^Chinese Department, Lingnan University, Hong Kong, Hong Kong SAR, China; ^3^College of Life Science, Sichuan Agricultural University, Yaan, China; ^4^School of International Studies, Wenzhou Medical University, Wenzhou, China

**Keywords:** educational psychology, literature education, literary situation, construction of the writing subject, teaching strategy

## Abstract

Educational psychology focuses on the laws of change in the knowledge, skills, and individual psychology of the educatees in the process of education and teaching. Writing teaching is a key and difficult point in literature teaching. Nowadays, it is common for students to be afraid and tired of writing in school literature education. In view of these problems, the present work optimizes the teaching mode of writing from the perspective of reconstructing the writing subject. Through literature research and interdisciplinary analysis, a questionnaire is designed to analyze the literary situation and the reconstruction of writing subjects in literary education. The questionnaire is aimed at three aspects, namely the hidden educational effect of teachers’ personality charm, the influencing factors of students’ psychology and students’ learning effect, and the influencing factors of psychology of the communication between teachers and students and teachers’ teaching effect. Then, the changes of students’ performance in literary class in these three aspects before and after using the teaching strategy of writing subject reconstruction are analyzed. Finally, the changes of students’ grades in the literary course are investigated. In this experiment, a total of 400 questionnaires were distributed, and a total of 389 questionnaires were collected. The survey results show that the number of students who feel the classroom atmosphere is active increases by 10%, the number of students who listen carefully and take notes increases by 7%, and 45% of students have improved their grades. Besides, after the implementation of the teaching strategy, most students change their attitude to the literature course, become more active, and significantly improve their motivation for learning. This study has a certain reference value for the analysis of literary situations and the reconstruction of writing subjects in literary education from the perspective of educational psychology.

## Introduction

Since the early 20th century, various literary theories have emerged endlessly, such as Russian Formalism, Anglo-American New Criticism, structuralism, and postmodernism. Literary research has gradually become cultural research, and the external environment of literary education has undergone great changes. There are mainly three aspects to discuss the problems existing in the current literature education. Firstly, literature education tends to be toolized. That is, literature is regarded as a tool for political education, moral cultivation, and knowledge and skills teaching (Chipchase et al., 2017, p. 31–42). Secondly, the environment of literature education is unduly market-oriented. With the development of the market economy, literary creation is affected by public interest and economic interests, and literature publications are increasingly catering to capital and the public (Coughlan et al., 2018, p. 496–506). The development of television, networks, and other media also provides literature publications with a broader communication platform. Thirdly, there is insufficient esthetic education. How to Read Literature suggests guiding readers to return to the basis of text reading through the analysis of words in literary works. These efforts give readers the key to opening the meaning of the work and help readers understand the unique significance of narrative techniques, portraits, and scene settings of different writers in the narration process (Jadon et al., 2018, p. 147–156). Besides, through reading literary works in different periods, readers can better understand the development process and objective laws of the times.

Educational psychology is an independent branch of psychology that combines psychology and education. Its research object is the psychological phenomena and laws of basic psychology of learning and teaching concentratedly reflected in the teaching process (Gray et al., 2018, p. 97–113). Literary education needs to understand the psychological characteristics of different students in different situations and master the laws of students’ psychological activities to see the problems of students and grasp the essence of students’ problems (Kahu and Nelson, 2018, p. 58–71). Only in this way can the literary education of students be timely, accurate, reasonable, and targeted, and achieve double efforts (Hilpert and Marchand, 2018, p. 185–202). The research object of educational psychology can be roughly analyzed from three levels (Ergas and Hadar, 2019, p. 757–797). First, in the process of teaching and learning, students’ own psychological development process is, actually, the focus of the learning process. From this perspective, when there is a certain research object of educational psychology, the core of the learner under education is the learning psychology and the learning and development law of intelligence, personality, ideology and moral, knowledge, and skills. Secondly, the teaching process includes the activities of teachers and students. The leading role of teachers is to give full play to the subjective initiative of objects. Educational psychology must also study various factors that can affect students’ learning to help students improve their learning enthusiasm. Students here are not only the object of education but also the subject of education. Thirdly, the teaching process in schools is, essentially, the process of interaction between teachers and students, during which the psychology and behavior of teachers and students change to achieve educational goals, which restrict and influence each other. This interaction between teachers and students is also included in the study of educational psychology. At present, there are a few research results on educational psychology in China. Some scholars use the constructivism theory of educational psychology to guide curriculum teaching in colleges and universities. These researchers believe that constructivism learning theory is related and appropriate to the current curriculum teaching reform in colleges and universities, and the series of views and ideas of the theory have profound implications for innovative education in curriculum teaching. Some researchers analyze the features of teachers, students, and teaching methods in college curriculum teaching from the perspective of educational psychology. They think that teachers should understand and grasp the psychological characteristics and laws of students in the teaching activities of colleges, and carry out teaching with definite aims by effective teaching methods under the consideration of actual situations. In summary, through the analysis of the current research status worldwide, a proliferation of scholars has realized the significance of educational psychology in college curriculum teaching. In a period of time, the research in this field will stay in the theoretical stage, and it will gradually turn to systematic investigation and empirical application combined with practice to promote the development of college curriculum teaching more effectively.

Through browsing related literature in this field, it is expected to analyze the possible circumstances and possible problems of literary research in the environment of educational psychology, and explore the changes of literary ecology and the current situation and future development direction of literary research. The innovation and the contribution of this work mainly lie in the combination of teachers, students, and the communication state between teachers and students, trying to investigate the influencing factors of educational psychology on the effectiveness of college literature education, deeply analyzing the internal hidden problems, and designing the corresponding teaching strategy based on educational psychology. Here, the practical problems of literary education are combined with educational psychology, which promotes the development of the transformation of research results into practical application. The teaching strategy of reconstructing the writing subject can help students better understand articles to improve students’ literary capacity.

In the next part of research approaches and procedures, related theories of educational psychology are elaborated, and the literary situation and reconstruction of writing subjects in literature education are deeply analyzed from the perspective of educational psychology. Then, literature research, comprehensive analysis, and questionnaire survey are selected as primary research methods. (1) The literature research method is to learn from relevant materials, such as newspapers, periodicals, papers, and books. Through the literature research method, researchers can understand the current research situation of the particular topic, form a general impression of the topic, understand the previous research results, and accumulate a substantial and feasible theoretical foundation for the topic research. (2) The comprehensive analysis method refers to analyzing and summarizing data. There are different understanding angles and views on the research on teaching, educational psychology, and the combination of two fields in academia. Therefore, it is essential to study, induce, and arrange plenty of papers for a convenient grasp of the existing research results. (3) The questionnaire survey method is a method of empirical research, and a survey means indirectly collecting research materials. The questionnaire survey method is conducive to objective analysis of the current teaching situation of literature courses and finding out the existing problems to put forward targeted solutions.

The practicability of the present work lies in revealing the possible correlation among college students’ literary writing style, literary writing anxiety, and literary writing achievement, and finding that the teaching strategy of reconstruction of writing subjects can transform the writing style. Moreover, the research content reveals that writing style is a factor causing writing difficulty and writing anxiety. This is conducive to understanding students’ attitudes toward different writing styles, writing anxiety, and writing achievements, which can better guide the teaching of literary writing.

The innovation of the present work lies in two points. On the one hand, through reading multitudes of relevant literature, it is concluded that writing style is a significant cause of writing anxiety. Therefore, based on the idea of subject reconstruction teaching, the writing subject is reconstructed to change the writing style, to alleviate students’ anxiety, and to improve the students’ writing performance. On the other hand, based on the innovative design of the questionnaire on the situation of Chinese literature teaching, it is found that the hidden educational effect of teachers’ personality charm has a great impact on the quality of classroom teaching.

The present work can be divided into five parts. The first part explains the development status of educational psychology, the reconstruction of the writing subject, and the research methods. The second part discusses research on literature education based on educational psychology. The third part designs a questionnaire on the teaching of Chinese literature. The fourth part makes an in-depth analysis of the relationship between the teaching strategy of reconstructing the writing subject and the progress of students’ academic achievement. The fifth part analyzes the survey results and draws the corresponding conclusions.

### Related Research on Literature Education and Educational Psychology

#### Research on the System of Literary Education

The specialized education with Chinese literature as the main content of education in higher education in China is called Chinese Literature Professional Education, mainly set up in literary colleges or Chinese departments, excluding public quality education such as College Chinese or Literary Appreciation. The inheritance of the important connotation of national culture in the cultural self-confidence advocated by the state cannot be separated from the professional knowledge of literature education. Meanwhile, the education and teaching of literature specialty also provide an important method and a path for the inheritance and shaping of national culture (Lippe and Carter, 2017, p. 402–408; Macfarlane and Tomlinson, 2017, p. 5–21). With the development of modern construction in China since the 20th century, Chinese literature education has fallen from the core position of the classical era to a special education. It also has formed a modern system characterized by the stratification of subject knowledge in the process of disciplinization (MacLeod et al., 2019, p. 426–448). The education of this discipline has encountered a disciplinary crisis in the current sociocultural context, including the fuzzy boundary of subject knowledge, the generalization of social functions, and the reduction of employment adaptability. Therefore, it is essential to comprehensively understand the content of the Chinese Literature Professional Education to systematically analyze the problems of Chinese literature education in the modern system and the hidden dangers and put forward corresponding countermeasures. The internal basis of the Chinese Literature Professional Education is the function of literature education (Matthews, 2018, p. 97–113).

At the beginning of the 20th century, the Chinese Literature Professional Education attracted the attention of many scholars and educators represented by Liang Qichao, Wang Guowei, Cai Yuanpei, Lu Xun, Hu Shi, Zhu Guangqian, and Zhang Qingbing, since it began to follow the autonomy of modern disciplines. They made quite academic and practical elaborations on the related issues of Chinese literature education. Especially in the literature research and teaching practice, they gradually constructed the basic knowledge system and professional teaching system of Chinese literature education (Patall, 2021, p. 1–19). For example, Liang Qichao stated that it is necessary to change the novel in a country first to change the citizens in his prose On the Relationship between Novels and Group Governance. This statement has a position not to be sniffed in improving the position of novels in the knowledge structure of Chinese literature education (Peters et al., 2019, p. 632–637). The slogan of “discussing literature education” proposed by Wang Guowei, Cai Yuanpei, and Hu Shi et al., advocates replacing religious thought with esthetic education to gradually exclude xiaoxue (the traditional Chinese philology) and classical Chinese semantics. These Chinese scholars insisted on proving the independence of literature with European theories and discussing the rationality of literature education so that Chinese literature education could gradually get rid of the fate of traditional literature and history education, and develop in the direction of discipline and specialization. The confluence of Hu Shi’s slogan of “Literary Revolution” and the educational law of “National Language Unity” in the early Republic of China opened the era of written vernacular Chinese in literary creation, and promoted new literature to the forum of literary professional education (Rotgans and Schmidt, 2017, p. 175–184). Lu Xun’s teaching principle of “Rejuvenation and Special Education” has created the road of literature education practice, combining in-class and out-class, reading and writing, and teaching and creation. Zhu Guangqian’s thought of “artistic life” emphasized the importance of literary esthetic education. The textbooks of literary theory written by Ma Zonghuo, Yi Qun, Cai Yi, and Zhang Qingbing, and the textbooks of modern and contemporary literature history written by Zheng Zhenduo, Qian Liqun, Cheng Guangdun, and Wang Qingsheng have laid a solid foundation for the stability and solidification of the knowledge content of Chinese literature education in different periods (Rowan et al., 2021, p. 112–158).

The situation of modern Chinese literature education is analyzed as follows. (1) There are problems caused by the configuration of the education and teaching of Chinese literature. At present, Chinese literature education is principally implemented in the Chinese language and literature department in colleges and universities, which has caused many disputes in academic circles. For a long time, literature education and Chinese education have been confused. Language courses account for a large proportion of the curriculum of the Chinese language and literature department. In the early stage, Chinese literature education was greatly influenced by the traditional concept that literature was the study of articles. The educators at that time believed that language courses are interrelated with literary theory and literary history, and they have their own knowledge systems. Besides, they all embodied the idea of language education as the basis of literature when carrying out literary research. This mixed phenomenon of education leads to the emphasis on the artistry of literature and the neglect of the application of Chinese literature in the process of teaching. (2) The purpose of specialized education of social function generalization is to cultivate professional talents. However, the civil administration management system was implemented in ancient China. By the time of the Republic of China, educators were determined to enlighten and awaken the public. Their purpose was to train patriots, and the curriculum content was mainly based on traditional Chinese classics. After the founding of the People’s Republic of China, the teaching of Chinese literature focused on literary foundation and theory. However, in that era of planned economy, the division of labor was not fine. Therefore, the training goal of Chinese literature specialty primarily lay in literary teaching and research, and the curriculum provision was also relatively extensive, involving theoretical knowledge of literature, management, news, and so on. (3) Employment adaptability is reduced. At present, the Chinese literature specialty is less popular among students, since graduates find it difficult to meet the needs of social development. With the increase of social demand for practical talents, the employment rate of graduates of applied majors, such as engineering, management, and medicine, is increasingly high. On the contrary, liberal arts graduates suffer hardship in finding satisfying jobs, and the employment rate is far lower than the national average employment rate. This is because, after the Opium War, the Western tool concept invaded China, and Chinese people began to seek to achieve maximum efficiency by the best means. This rationalism led to the profit-seeking behavior of Chinese universities. Moreover, the phenomenon of social “secularization” is serious, resulting in the loss of humanistic spirit. Consequently, majors of Liberal Arts and Sciences cannot bring obvious material benefits, so it is labeled useless (Kneebone, 2017, p. 71–74).

#### Research From the Perspective of Educational Psychology

Educational psychology is the product of the combination of psychology and education with a history of 100 years. It is necessary to determine the research object of educational psychology to strictly define educational psychology (Sanderson and Brewer, 2017, p. 65–71). The research objects of educational psychology are roughly divided into three categories by different scholars. First, some scholars equated the research object of educational psychology with the research object of pedagogy. Second, some scholars equated their research objects with those of general psychology (Schindler et al., 2017, p. 1–28). For example, Edward Lee Thorndike stated that the research object of educational psychology was the study of human nature and the corresponding change in human nature in his representative work Introduction to Educational Psychology. Third, other scholars simply regarded their research objects as those of behavioral science. The Education: The complete Encyclopedia published in the United States points out that educational psychology mainly studies the behavior of the educatee in the process of education, and generally, the research object is also identified as a discipline closely related to the relationship between students’ learning and teachers’ teaching. The other definitions are vaguer and inaccurate, which are not to mention here (Lange and Dewitte, 2019, p. 92–100).

The researchers defined and discussed the reconstruction of writing subjects in literary education, and summarized the broad impact of related research (Matthews and López, 2020, p. 101878). The researchers believed that political education was carried out through formal courses, infiltration of various disciplines, practical teaching, psychological counseling, employment education, and other forms and methods, and it took moral education as the center to instill moral education ideas into the activities of the school (Lewicki and Shooman, 2020, p. 30–43).

Although there are small differences in the definitions of educational psychology, there is a very obvious common point, that is, to take the psychological law of the subject in the process of education as the research object of educational psychology. Although the words with limited modification such as “teaching” and “learning” appear in the definitions of some researchers, the teaching process is still the most important thing that they cannot ignore. The behavior of “teaching” or “learning” is originally the behavior of the teaching or learning subject. Psychological laws only exist in the subjects of behaviors rather than in the subjects’ behaviors. In other words, teachers and students have psychological rules only in the teaching process. Therefore, educational psychology should take “the psychological activities and psychological laws of students and teachers in the teaching process, and the psychological laws of teachers and students when they interact and influence each other: as the research object. Correspondingly, “educational psychology is a science that studies the students in the learning situation, the teachers in the teaching situation, the psychological activities and psychological laws of these students and teachers when they interact with each other.”

#### Research on Literature Education Based on Educational Psychology

**FIGURE 1:**
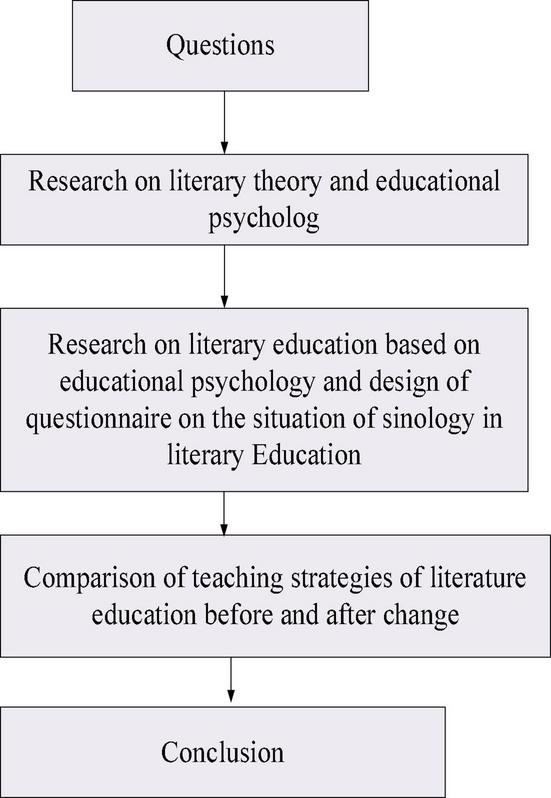
Research methods and procedure.

Students of four colleges were selected as objects of the questionnaire survey to further understand the actual situation of the application of educational psychology in literature teaching in colleges and universities to find effective teaching strategies to improve the effectiveness of literature education. The teaching level of the four universities is, basically, the same, and the number of students chosen from each school was the same. In the survey, SPSS 26.0 was used for data analysis, and the hardware configuration was Windows 1064-bit system. SPSS 26.0 is a leading data mining and statistical analysis software in the world, which can provide users with the most perfect core functions of data analysis, including abundant machine learning algorithms, text analysis, open-source scalability, integration with big data, and seamless deployment to applications. The questionnaire involves the hidden educational effect of teachers’ personality charm, the influencing factors of students’ psychology and students’ learning effect, and the influencing factors of psychology of the communication between teachers and students and teachers’ teaching effect. In this experiment, 400 questionnaires were distributed, 389 valid questionnaires were recovered, and the effective rate was 97.25%. The number of students, teachers’ teaching ability, and students’ learning ability of the four universities selected here were very similar. Meanwhile, the random sampling method was used for students of four grades in various majors, including water supply and drainage, literature, energy, information engineering, English, Chinese language, law, and economics. Therefore, this survey can basically ensure the rationality and comprehensiveness of the respondent, which provides a scientific and effective basis for the in-depth study and analysis of the impact of educational psychology on the effect of literary education in colleges and universities. There are various reasons for the weak effectiveness of literature education in universities, but many researchers generally believe that the most important factor is still teachers and students. It is hoped that the questionnaire survey based on the research of educational psychology to study literature education in colleges and universities can improve the effectiveness of literature education closer to reality. Figure 1 illustrates the research procedures of the present work.

#### Design of the Questionnaire on the Literary Situation in Literary Education

The three aspects of educational psychology were used here to analyze the psychological and educational effects of college literature teachers, including the teaching efficacy, personality charm, and teachers’ prestige.

First, teachers’ personality charm can produce hidden education effects. The personality charm of teachers’ personality characteristics will produce power influence in the teaching process, which is an implicit way of education. As the main body of literature education in colleges and universities and a living carrier, literature teachers will naturally exhibit their knowledge, demeanor, ideology, and morals, etc., in the teaching process, imperceptibly affecting the students and thus creating image effects. Therefore, a questionnaire is designed for the analysis of teachers’ personal charm from the aspects of image charm, literary quality, moral quality, and professional quality.

Second, students’ internal psychology influences their learning effect. The psychological problems of contemporary college students are increasing in life and learning. In the new era, literature professional education in colleges and universities is facing new and difficult challenges. Among them, the simplest and the most direct problem is reflected in the psychological problems and mental health of college students. It is essential for college students to master psychological activities in their learning process to face learning with motivation. Therefore, the concepts of personality tendency and self-consciousness in the field of educational psychology are the two starting points for the study and analysis of college students’ learning psychology and its influence on learning effect.

Third, the teaching effect is affected by psychological factors in the process of communication between teachers and students. As the main body of the teaching process, teachers and students are fully playing their own roles, which directly affects the process and effect of content produced by literary education in the interaction. Modern education theory believes that both teachers and students are indispensable. In teaching activities, the relationship between teachers and students, as well as the relationship between students, can have a great influence on the process of communication. Compared with the relationship between other subjects and objects, the relationship between teachers and students is the most basic and important one in literary education and teaching activities. This relationship is relatively special, and thus has a significant impact on the process of communication between teachers and students. The essence of the teaching process is the interaction between teachers and students. There is a strong humanistic concern and Marxist educational emotion in the process. Besides, the teaching goal of literature is achieved through the interaction and psychological integration between teachers and students. Therefore, harmonious communication between teachers and students in teaching activities is indispensable for high effectiveness and high quality of literature education in colleges and universities. The mutual individual attraction or intergroup attraction between teachers and students can enhance the development of teacher-student interaction on the foundation of equal and democratic relations between teachers and students, which, ultimately, has a huge impact on the teaching effect.

#### Literature Teaching Strategy of Reconstructing the Writing Subject

The subject was not unique to human beings before modern times. The concept of “subject” includes animals, plants, stones, and natural ecology. By improving their status and value, human beings have monopolized the concept of “subject” in modern times and naturally became the most basic and core subject in the world. Correspondingly, all the methods of existence and the basis of truth in the world become human beings when they are regarded as the real subject (Shubnikova, 2018, p. 552–565). The principle of human subjectivity regards human beings as the origin, center, and destination of the world to show the spirit of anthropology, anthropocentrism, and humanism, which prove the principle of human subjectivity (Slaby, 2017, p. 235–256). The concept of subjective metaphysics began from Plato and Aristotle, and entered the establishment stage from the Middle Ages to Descartes’s times, finally becoming mature in Hegel’s times (Stroe, 2020, p. 103–329). Therefore, anthropology observing the world from the human perspective has been born since then. In the process of humanitarianism, humanism, and anthropocentrism, human metaphysical subjectivity is truly determined (Takkal et al., 2018, p. 136–142). Therefore, all philosophies, truths, and world outlooks revolve around human beings, such as the generation of anthropology and anthropocentrism. In Heidegger’s view, the whole human history is nothing more than a process of constantly constructing and deepening metaphysical subjectivity, while gradually forgetting “Dasein (being in the world)” (Uerz et al., 2018, p. 12–23). “Dasein” abandons human beings, while human beings can forget “Dasein.” Is not this the most essential crisis reflected in modern times? This is also Heidegger’s point of view (Walsh et al., 2020, p. 102748). Although the metaphysical subjectivity theory of humans is bound to end in the future world, it does not represent a simple cessation, extinction, or hopelessness, but may be a form of completion of all possible accomplishments (Whitehead, 2020, p. 159–175). Therefore, it is better to overcome or surpass it as much as possible. Researchers believe that human nature and humanity in metaphysics are also part of the being, that is, human beings in the traditional metaphysics are natural and biological. In fact, the affirmation of human beings is Heidegger’s pursuit, aiming at a more profound “reconstruction” or “regaining” of human nature.

The reason for using this research method is that, in the 21st century, with the continuous development of science and technology, there are new requirements for college students’ literature education. In the teaching process of literature courses, teachers should not only teach theoretical knowledge but also pay attention to cultivating students’ literary literacy and promoting students’ development in all aspects. Teachers are required to innovate the teaching mode on the basis of traditional teaching, infiltrate the core literacy of literature into classroom teaching, and improve teaching efficiency and quality. The research on the literary situation and the reconstruction of writing subjects in literary education can help students understand literary works, help students improve their grades, and meet the actual needs of college literary education.

#### Relationship Between the Teaching Strategy of Reconstruction of Writing Subjects and the Progress in Academic Performance of Students

Writing plays an extremely significant role in language learning and language acquisition. Writing ability is affected by many factors, among which emotional factors have a critical impact on writing. Moreover, anxiety is a vital variable in emotional factors, which is worthy of in-depth study. Anxiety is an unpleasant emotional state, accompanied by subjective feelings, such as tension, anxiety, and worry. It can be divided into personality anxiety, state anxiety, and situational anxiety. Language anxiety is a kind of situational anxiety that students experience when using their unskilled foreign language. Anxiety can create obstacles in the whole process of language learning and have a great negative impact on language learners. There are many studies on foreign language anxiety, and the research on writing anxiety began in the late 20th century. Foreign and domestic existing works on writing anxiety show that there is a negative correlation between writing anxiety and writing achievement. Under the framework of Krashen’s affective filtering theory and information processing theory, the research results of other researchers are analyzed here to explore the relationship between writing style, writing anxiety, and writing achievement. The results preliminarily confirm that writing style is one of the causes of writing anxiety, and there is a significant positive correlation between writing anxiety and writing achievement. The reconstruction of the writing subject can change the writing style, reduce students’ writing anxiety, and improve students’ writing performance. In the process of writing teaching, teachers should also offer students with language knowledge and writing skills required for different styles of writing to ameliorate the students’ writing anxiety.

#### Reliability and Validity Analysis of the Questionnaire

Here, SPSS 26.0 was used to analyze the reliability of the questionnaire, and Cronbach’s alpha coefficient was 0.804, which is larger than 0.7, indicating good reliability. Table 1 summarizes the results of reliability statistics.

**TABLE 1:** Reliability statistics.

Cronbach’s alpha coefficient	Cronbach’s alpha coefficient based on standardized terms	Number of terms
0.824	0.803	9

Then, the KMO test and the Barlett test were performed on the questionnaire by SPSS 26.0 for factor analysis to investigate the validity of the questionnaire. The KMO value after the test was 0.657, greater than 0.6, so each question in the questionnaire was suitable for factor analysis, with good construct validity. Table 2 illustrates the results of the KMO test and the Bartlett test.

**TABLE 2:** Results of the KMO test and the Bartlett test.

Kaiser-Meyer-Olkin of sampling adequacy	Measurement	0.657
Sphericity test of Bartlett	Approximate chi-square	142.178
	df	57
	Sig	0.000

Through the above analysis, the questionnaire has good reliability and validity, and the survey data of students are reliable.

### Results Before and After the Change in Teaching Strategies of Literature Education

#### Survey Results of the Literary Situation in Literature Education

**FIGURE 2:**
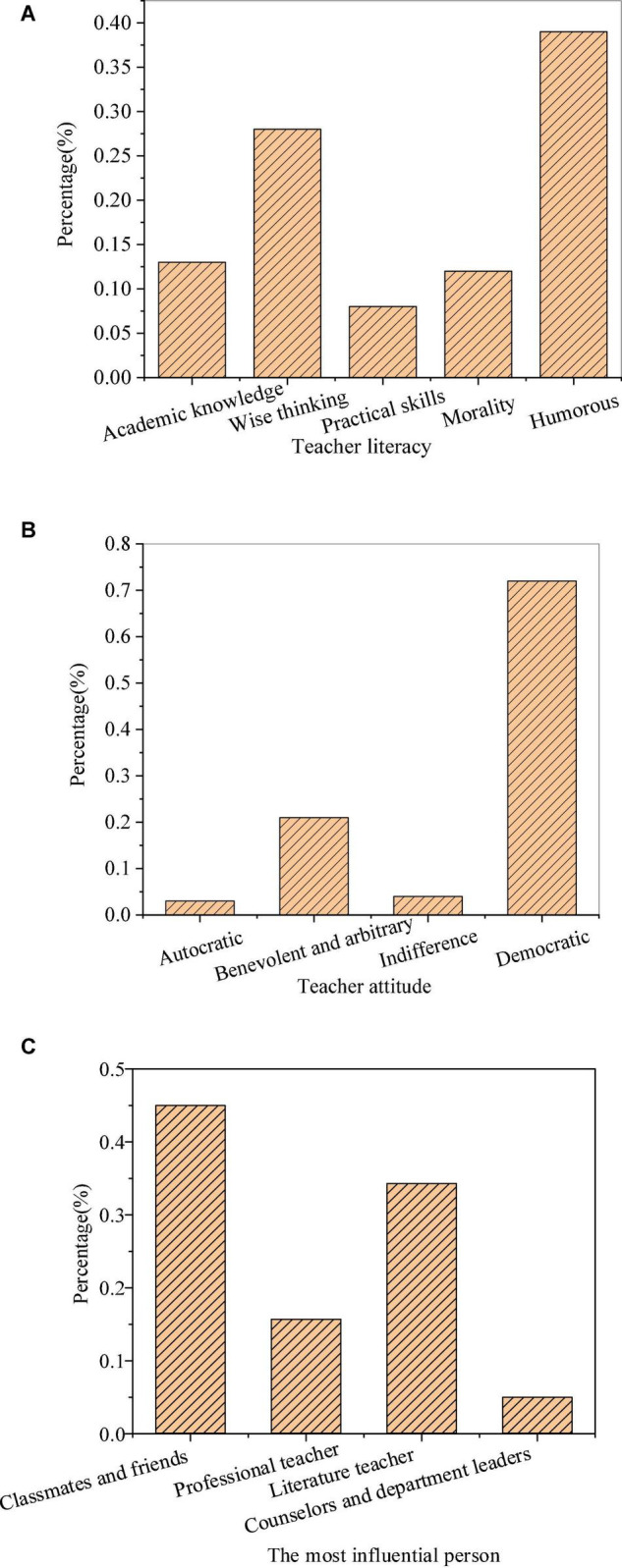
Hidden education effects of teachers’ personality charm (**(A)**: teachers’ literacy; **(B)**: teachers’ attitude; and **(C)**: the most influential person in literature learning).

Figure 2 shows the hidden education effects of teachers’ personality charm.

The survey found that 38.9% of the students had preferred humorous teachers, 27.9% of the students preferred intelligent and ideological teachers, and academic knowledge, moral style, and practical skills were less popular with the students. In terms of teachers’ attitudes, 71.5% of the students liked democratic teachers, followed by benevolent and arbitrary teachers supported by 21.5% of the students, while autocratic teachers and cold teachers had little support from the students. In the survey of teachers’ teaching efficacy, the students thought that the most influential persons to their literary literacy were classmates and friends, accounting for 46.4%, followed by literature teachers, accounting for 33.1%. In terms of the charm of teachers’ academic knowledge, that is, professional quality, teachers with extensive knowledge and profound theoretical accumulation can have common topics with students to better maintain the far-reaching impact on students. The reason why teachers’ prestige has such a great influence is that students will take teachers with prestige as their own examples. In this case, teachers’ praise or criticism of students can more arouse students’ corresponding emotions and greatly reduce the students’ rebellious emotions toward curriculum teaching. Meanwhile, teachers with prestige not only can more easily convince students of the authenticity of their teaching contents and the correctness of their guidance but also more rapidly transform the teachers’ requirements into the students’ needs. The teachers can enhance students’ enthusiasm and initiative in learning, and improve the students’ acceptance of teaching to significantly improve the educational effect. Teaching efficacy will indirectly affect the teaching effect by affecting teachers. Teaching efficacy will affect teachers’ emotions in teaching, organization and control of teaching activities, and application of teaching strategies. Literature teachers with high teaching efficacy will have a high sense of security and self-confidence in the teaching process. They will show a positive attitude and emotion in teaching, and can effectively choose teaching methods and strategies. Moreover, they will also be willing to spend more time in teaching organizations to create a relaxed and democratic classroom atmosphere. Therefore, teachers’ teaching efficacy has become a critical factor affecting teaching quality.

**FIGURE 3:**
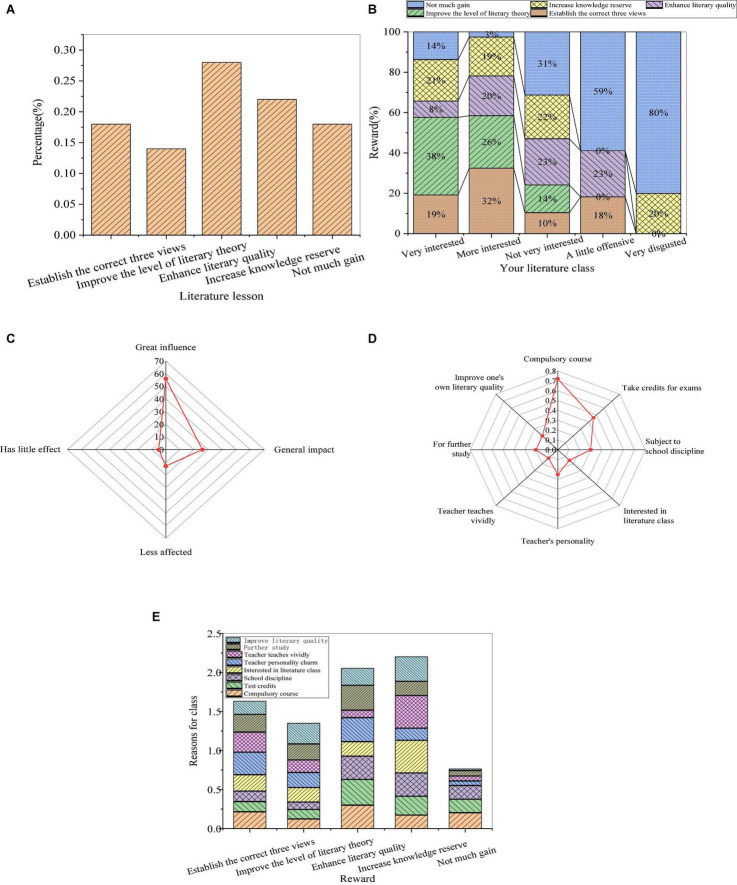
Self-assessment of the teaching efficiency (**(A)**: acquisition from the course; **(B)**: relationship between the acquisition and attitude; **(C)**: influence of learning attitude on learning effect; **(D)**: learning motivation; and **(E)**: relationship between the learning motivation and reasons for the class).

The assessment of the educational effects of a roughly systematic study of literature on themselves is shown in Figure 3.

From Figure 3, most of the students enhance their literary quality in the literature course, accounting for 29.4%, followed by increasing knowledge reserve, occupying 21.3% of the students. Apart from the students who can establish the correct three views or improve the level of literary theory, more than 10% of the students still believe that they have no gain. Through results of the relationship between the acquisition and learning attitude of students in the literature course, students with more interest benefit more from the literature course, while the less interested students obviously have less acquisition. In the survey of the influence of students’ attitude on the learning effect, more than half of the students thought that their learning attitude had a great influence on the learning effect. Meanwhile, for learning motivation, the literature course is a compulsory course for about 75% of the students. Therefore, external constraints rather than autonomic learning are the reason for most students taking literature courses with a few learning needs. From the results of the relationship between learning motivation and acquisition, among the students who have no gain in the literature course, 86.8% are students who are compulsory to take the literature course, and 52.6% are students who want to gain credits. However, students who benefit little from the literature course with the motivation of enhancing their literary quality occupy a small perception of the total students gaining little in the literature course.

From the above data, students have serious psychological problems in the process of learning literature. It is difficult for students to raise their interest in learning literature. Besides, external motivation is much higher than internal motivation, and they do not realize the real function of literature courses. Some students deny the setting of literature course in cognition, and the need for consciousness of learning literature course shows passivity. In the research of personality psychology, educational psychologists have shown that personality tendency is the dynamic structure of personality, and need is the source of enthusiasm of organism activities. Evidently, the need and the motivation for the external world are the initial motivation source of college students’ learning enthusiasm. Therefore, to improve the initiative and enthusiasm of learning literature, students should cultivate their own awareness of needs, correct their cognitive deviations, and produce correct learning motivation to form good learning habits.

**FIGURE 4:**
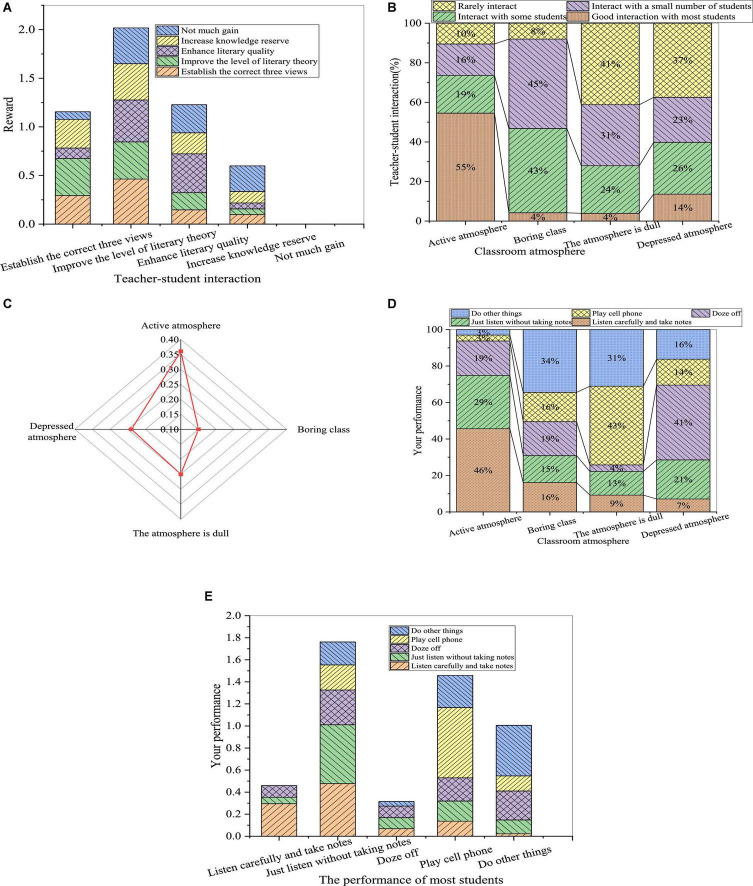
The influence of the interaction between teachers and students on the teaching effect (**(A)**: relationship between the interaction and acquisition; **(B)**: relationship between the classroom atmosphere and the interaction; **(C)**: the classroom atmosphere; **(D)**: relationship between the classroom atmosphere and students’ performance; and **(E)**: inter-effect of students’ performance).

The influence of the interaction between teachers and students on the teaching effect is shown in Figure 4.

From Figures 4A,B, most of the teachers who can create a more active classroom atmosphere for students in the literature course are those interacting well with most of the students, among whom only a few students have little acquisition. If the teacher only interacts with some students or rarely interacts with students, the teaching atmosphere will make students feel dull or depressed. Therefore, literature teachers should actively interact with students in teaching activities to mobilize the initiative of the students and finally create a psychologically interactive and relaxed classroom atmosphere. The corresponding atmosphere is conducive to the development of education and teaching activities to ultimately achieve the purpose of good teaching. According to students’ evaluation of the classroom atmosphere, about 37% of the students think that the classroom atmosphere is active, and 50% of the students totally think that the atmosphere is dull or depressed. Furthermore, most students in an active classroom listen carefully and take notes, indicating that the classroom atmosphere has a significant influence on the teaching effect of literature. By comparing the performance of other students with the performance of the respondents, the respondents are affected when most of the students in the literature class listen carefully and take notes. This shows that the performance of surrounding students has an influence on the performance of students in the literature course. Therefore, teachers should create an active classroom atmosphere and pay attention to the mutual psychological influence of students.

From Figure 4C, about 37 % of the students think that the classroom atmosphere is active, and the sum of the number of students who think that the atmosphere is dull or depressed is about 50%. Besides, most of the students in the active classroom will listen carefully and take notes, so the classroom atmosphere has a significant influence on the effect of literature teaching. Through Figures 4D,E, comparing the classroom performance of other students and the classroom performance of the respondents themselves, when most students listen carefully to lectures and take notes in the literature classroom, the respondents themselves will listen carefully to lectures and take notes. Besides, the respondents who just listen without taking notes play mobile phones, or do other things will also be affected. Therefore, the learning behavior of students in the surrounding groups will affect the learning behavior of the students themselves. Therefore, teachers should be good at creating an active classroom atmosphere and pay attention to the psychological influence of peers. From the above analysis, the relationship between teachers and students in the teaching process will directly affect the classroom atmosphere, indicating the relationship between individual psychology and group psychology, which is also an indispensable part of handling the relationship between teachers and students in the teaching process. In summary, the development of the relationship between teachers and students plays an important role in the classroom atmosphere in college teaching activities, and the psychological relationship between individuals and groups is also a vital factor in dealing with the relationship between teachers and students in the teaching process. The relationship between teachers and students based on equality and democracy can enhance the mutual attraction between teachers and students, and between individuals and groups, arouse the desire for communication between teachers and students, and affect the teaching effect.

#### Effects of the Teaching Strategy of Reconstructing the Writing Subject

**FIGURE 5:**
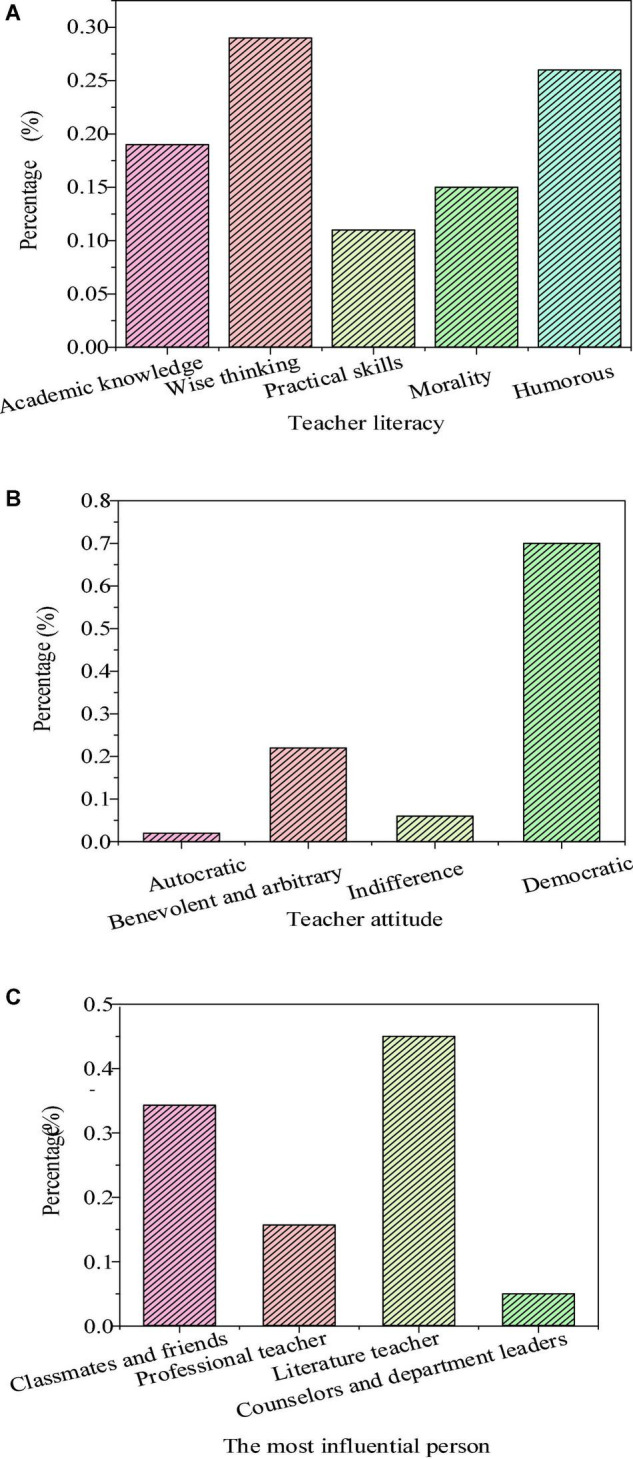
Hidden education effects of teachers’ personality charm **(A)**: teachers’ literacy; **(B)**: teachers’ attitude; and **(C)**: the most influential person in literature learning.

The teaching effect is significantly improved by the teaching strategy of reconstructing the writing subject. The influence of teachers is shown in Figure 5.

The teaching effect is significantly improved by the teaching strategy of reconstructing the writing subject. Through the survey data, the percentage of students who prefer academic teachers increases by 6%, while the number of students who like domestic teachers almost remains the same. Meanwhile, there is also a significant change in the survey results of teachers’ teaching efficacy. Most of the students regard the literary teacher as the person who has the greatest influence on their literature quality, increasing by 11%. In teaching, if teachers want to make students better recognize and accept their views, they must build strong prestige in students’ minds. The prestige of teachers will lead to an effect of model education for students, affect teachers’ teaching enthusiasm and teaching attitude, and result in a huge impact on teaching effectiveness.

**FIGURE 6:**
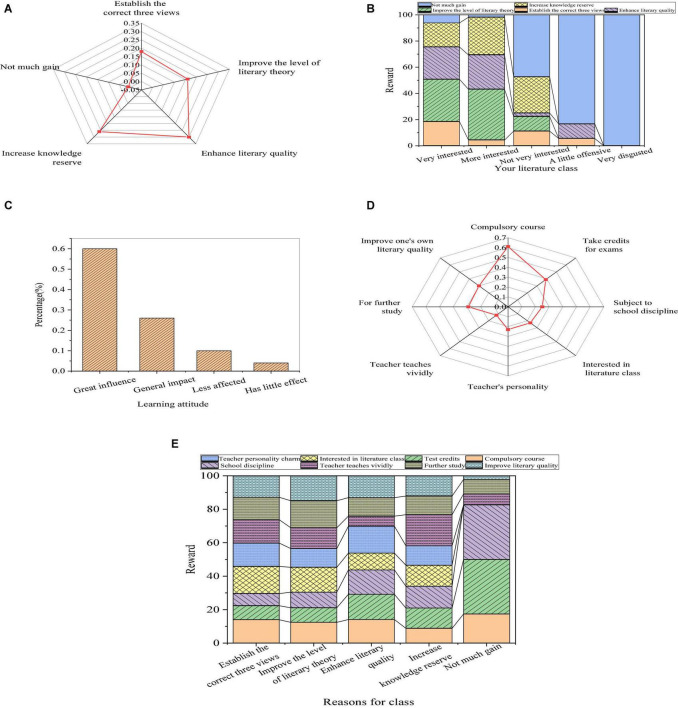
Influence of students’ factors on the teaching effect of reconstructing the writing subject (**(A)**: acquisition from the course; **(B)**: relationship between learning attitudes with the acquisition; **(C)**: learning attitudes; and **(D)**: relationship between the motivation and reasons for the class; **(E)**: relationship between the learning motivation and reasons for the class).

Figure 6 shows the influence of students’ factors on the teaching effect after using the teaching strategy of reconstructing the writing subject.

From Figure 6, the literary quality cultivation and knowledge reserve of students in different universities increased by 2 and 4%, respectively. Besides, the number of students who are very interested in literature courses increases by 5%. Moreover, the number of students who think their learning attitude has a great impact on literature learning increases by 4%, and more students are motivated to improve themselves rather than forced to study. It demonstrates that, after using this strategy, students learn less for external motivation, increase literature learning for internal needs, and gradually narrow the gap between learning effect and teaching objectives. Therefore, the research on the literary situation and reconstruction of writing subjects based on educational psychology reported here can effectively improve students’ achievement and interest in literature.

**FIGURE 7:**
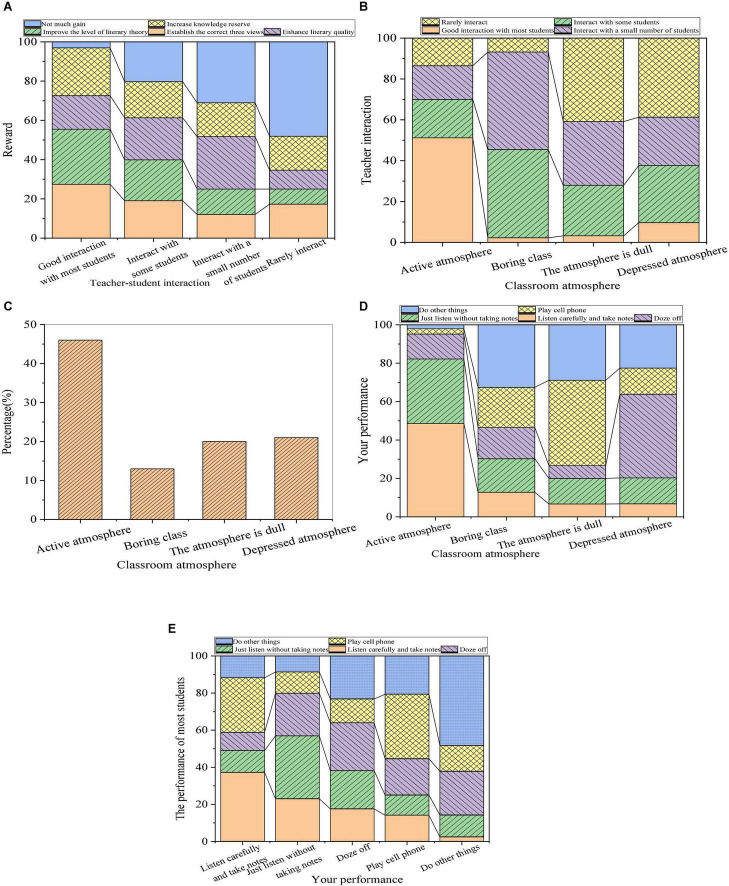
Influence of the teacher-student interaction on the teaching effect (**(A)**: relationship between the interaction and acquisition; **(B)**: relationship between the interaction and the classroom atmosphere; **(C)**: the classroom atmosphere; **(D)**: relationship between the classroom atmosphere and students’ performance; and **(E)**: inter-effect of students’ performance).

The influence of teacher-student interaction factors after the teaching strategy of reconstructing the writing subject is presented in Figure 7.

By comparing the results of Figures 4, 7, it is obvious that students’ acquisition in the class is positively correlative with the teacher-students interaction, and the classroom atmosphere is more active with more teacher-student interaction. Students increase by 10% who feel that the classroom atmosphere is more active. Meanwhile, students listening carefully and taking notes increase by 7%. The total number of students studying hard in the literature class increases by 8%.

**FIGURE 8:**
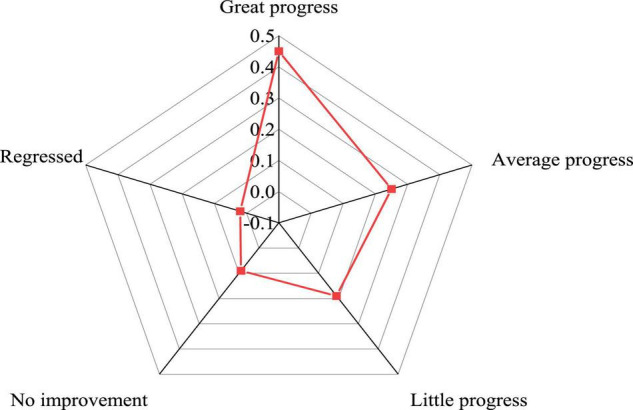
Improvement of students’ academic performance after changing the teaching strategy.

Figure 8 shows the effect of the teaching strategy of reconstructing the writing subject on the improvement of students in literature.

From Figure 8, 45% of the students think that their grades have made great progress, 25% think that their progress is moderate, and 19% think that their progress is small. Although there are students with little progress, most of them have made some progress in literature learning through changing the teaching strategy. In literature learning in colleges and universities, students should change their attitude toward literature teaching, correct their own personality deviation, and cultivate correct self-consciousness and self-behavior. Meanwhile, they need to strive to overcome learning obstacles, master scientific learning methods, become strategic learners, actively absorb the scientific content of literature teaching, and enrich their own theory foundation.

The experimental results demonstrate that, if the teacher gives students reasonable expectations in the teaching process, after experiencing the teacher’s attention and encouragement, students’ self-esteem, self-confidence, self-love, and self-improvement psychology will make them strict with themselves according to the norms. Besides, they will face the learning life with a positive passion to achieve the desired effect of the teacher. College teachers should pay attention to the Rosenthal effect, integrate teachers’ expectations into teaching, evaluate students comprehensively and objectively, pay attention to each student as much as possible, give them reasonable expectations, and lead them to the right path of ideological development.

## Conclusion

With the promotion of the reform and demonstration of the education industry, educational psychology plays an increasingly prominent role in education and teaching. Therefore, from the perspective of educational psychology, the current situation of Chinese literature education and the application of the reconstruction of writing subject to writing teaching are analyzed through literature research, interdisciplinary analysis, and a questionnaire survey. This questionnaire survey considers three aspects, namely the hidden educational effect of teachers’ personality charm, the influencing factors of psychology and learning effect of students, and the communication psychology between teachers and students and influencing factors of teachers’ teaching effect. Based on the results of the questionnaire, the present work analyzes the changes in students’ performance in these three aspects in literature class before and after the implementation of the teaching strategy of writing subject reconstruction. The survey results demonstrate that, after the implementation of the subject reconstruction teaching strategy, most students’ attitude toward literature curriculum has become more positive, and their learning motivation has been significantly improved. In addition, more students are willing to listen carefully and take notes. In addition, nearly half of the students think that their grades have made great progress. A few students think they have made average progress, and a few students think they have made little progress. Facts have proved that it is essential to use educational psychology to analyze the literary status of literary education and reconstruct the writing subject, which can effectively improve the teaching effect. The innovation and the practicability of this work lie in finding effective teaching methods in the process of literature teaching to alleviate students’ writing anxiety and help students improve their literary achievements.

However, there are still some deficiencies here. Due to the limited time, the survey sampling data are not sufficient. Future research will expand the amount of data through more students and a more comprehensive questionnaire design to find the internal relationship between students’ psychology and literature learning. Moreover, only writing style is considered in the study of the relationship between students’ writing anxiety and writing achievement, while other factors are not explored. The follow-up work will take writing tests, questionnaires, and individual interviews as research methods to investigate the correlation between students’ writing anxiety and writing strategies more deeply based on the affective filtering hypothesis and social interdependence theory.

## Data Availability Statement

The raw data supporting the conclusions of this article will be made available by the authors, without undue reservation.

## Ethics Statement

The studies involving human participants were reviewed and approved by Wenzhou Medical University Ethics Committee. The patients/participants provided their written informed consent to participate in this study. Written informed consent was obtained from the individual(s) for the publication of any potentially identifiable images or data included in this article.

## Author Contributions

All authors listed have made a substantial, direct, and intellectual contribution to the work, and approved it for publication.

## Conflict of Interest

The authors declare that the research was conducted in the absence of any commercial or financial relationships that could be construed as a potential conflict of interest.

## Publisher’s Note

All claims expressed in this article are solely those of the authors and do not necessarily represent those of their affiliated organizations, or those of the publisher, the editors and the reviewers. Any product that may be evaluated in this article, or claim that may be made by its manufacturer, is not guaranteed or endorsed by the publisher.
